# Interactions between Active Ingredient Ranitidine and Clay Mineral Excipients in Pharmaceutical Formulations

**DOI:** 10.3390/ma13235558

**Published:** 2020-12-06

**Authors:** Lijuan Wang, Xisen Wang, Libing Liao, Qingfeng Wu, Hui Yin, Zhaohui Li

**Affiliations:** 1 Beijing Key Laboratory of Materials Utilization of Nonmetallic Minerals and Solid Wastes, National Laboratory of Mineral Materials, School of Materials Science and Technology, China University of Geosciences, 29 Xueyuan Road, Beijing 100083, China; 2004011841@cugb.edu.cn; 2 Chemistry Department, University of Wisconsin–Parkside, Kenosha, WI 53144, USA; wangx@uwp.edu; 3 School of Physics and Optoelectronic Engineering, Yangtze University, 1 Nanhuan Road, Jingzhou 434023, China; wqfscience@aliyun.com; 4 Key Laboratory of Arable Land Conservation (Middle and Lower Reaches of Yangtse River) Ministry of Agriculture, College of Resources and Environment, Huazhong Agricultural University, Wuhan 430070, China; yinhui666@mail.hzau.edu.cn; 5 Geosciences Department, University of Wisconsin-Parkside, Kenosha, WI 53144, USA

**Keywords:** cation exchange, clay minerals, desorption, excipients, ranitidine, sorption

## Abstract

Excipients play an important role in pharmaceutical formulations. Many clay minerals, because of their large specific surface area and inert behaviour in reactions with active ingredients, are commonly used as excipients. In this study, the uptake of ranitidine (RT), the active ingredient of Zantac, on and released from palygorskite (Pal), kaolinite (Kao), and talc was evaluated under different physicochemical conditions. The results showed that the uptake of RT on these minerals was limited to the external surface areas only. Cation exchange and electrostatic interactions were responsible for the RT uptake on Pal and Kao, resulting in a monolayer sorption. In contrast, multilayer RT uptake was found on the talc surfaces. Under different desorbing conditions, significant amounts of sorbed RT remained on the solid surface after 5 h of desorption. The results suggest that the sorptive interactions between the active ingredients and the excipients may not be neglected in pharmaceutical formulations, should these minerals be used as additives and/or excipients.

## 1. Introduction

Inactive ingredients and excipients are used extensively in the pharmaceutical industry. They can be divided into synthetic and natural compounds. Although synthetic compounds are commonly used in modern pharmaceuticals because of their higher purity and less interactions with active ingredients, natural Earth materials, particularly clay minerals, used either as active ingredients or as excipients, are also common practice in the pharmaceutical industry [1,2,3].

Clay minerals are divided into 1:1 layers and 2:1 layers based on the stacking of the Si–O tetrahedral sheet and Al–O octahedral sheet, with the 1:1 layered clay minerals made of one tetrahedral sheet and one octahedral sheet. In contrast, the 2:1 layered clay minerals are made of one Al–O octahedral sheet sandwiched in between two Si–O tetrahedral sheets. Further division of the clay minerals is based on whether the octahedral sites are filled with trivalent Al^3+^ or divalent Mg^2+^, with the former being dioctahedral and the latter being trioctahedral. Types of interlayer cations and their hydration states affect the swelling properties of clay minerals and their cation exchange capacity (CEC). Montmorillonite (MT) group minerals, because of their swelling property, large specific surface area (SSA), and high CEC values, have been investigated extensively for their pharmaceutical use. Serving as an active ingredient, MT has been fabricated into tablets in order to control bowel movement in diarrhea patients because of its high water sorption and swelling property.

Talc is a trioctahedral 2:1 layered phyllosilicate without interlayer cations. Talc has been widely used in conventional dosage forms, like tablets, pills, and capsules, as a pharmaceutical excipient because of its physicochemical, physiological inert, and inexpensive nature [4]. Adding talc achieved a slow release of carbamazepine, able to release 70% of the drug over a range of 0.5–3.0 h [5]. However, interactions between talc and active ingredients have been reported in recent studies. Talc has a strong affinity for antibiotic ciprofloxacin (CIP) with a sorption capacity of 0.74 mg/g [6]. The sorption capacity of atenolol (AT) on talc was 11 µmol/g, or about 3 mg/g [7]. Similarly, the uptake of ranitidine (RT) on talc was 15 µmol/g, or about 5 mg/g [8]. An uptake of chlorpheniramine (CP) as high as 11 µmol/g was reported for talc [9]. The sorption of sulfadiazine on talc followed a linear isotherm with a distribution coefficient of 5.6 L/kg [10]. On the opposite side, no interaction was found between talc and nateglinide [11]. These studies demonstrate that further work is still needed in order to study the effect of sorption of drugs on talc [12].

Kaolinite (Kao) is a 1:1 layered dioctahedral phyllosilicate. Kao could serve as a suitable inexpensive excipient, and the interaction between Kao and drugs may be useful in the design of modified drug delivery systems [13]. The most important functionalities of Kao in pharmaceutical formulation are as a diluent, binder, disintegrant, pelletizing and granulating, amorphizing, particlefilm coating, emulsifying, and suspending agent [13]. Similar to talc, 5-fluorouracil, sorption on Kao could be on the external surfaces and in the interlayer spaces after being modified with dimethyl sulfoxide [14]. An uptake of CIP as high as 6% was found on Kao [15]. Under low pH conditions, such as in the stomach fluidal cases, benzodiazepine diazepam was found to sorb on Kao [16]. In the presence of Kao, chloroquine partitioning into the buccal membranes was decreased, which would reduce its effectiveness as an anti-rheumatic or anti-malarial drug if taken together with Kao [17]. The incorporation of Kao into the formulation of chloroquine and chlorpheniramine tablets resulted in a significant reduction in the amount of the active drugs released into the solution [18].

Palygorskite (Pal), also called attapulgite, is a 2:1 layered fibrous dioctahedral phyllosilicate. Because of its large CEC value, the sorption of cationic drugs on Pal is much higher in comparison with talc and Kao. The sorption of CIP on Pal could reach a capacity of 160 µmol/g [19]. Similarly, the uptake of RT on Pal reached 156 µmol/g [20]. The interaction between Pal and ethambutol (ETB) resulted in approximately 15.9% sorption via hydrogen bonding between the amino and hydroxyl groups of ETB and the O atoms of the mineral surface, and the composite ETB/Pal also provided a better physical stability with reduced hygroscopicity [21]. Isoniazid (INH) is one of the most effective first-line drugs in the treatment and prevention of tuberculosis (TB) [22]. Using Pal as a nanocarrier, the uptake of INH on Pal could reach 13 mg/g under pH 2, and more than 60% was released in the intestinal medium (pH 6.8 and pH 7.4) [22]. No interactions were found between rifampicin and isoniazid and Pal, and the presence of Pal did not interfere with the dissolution of both drugs, suggesting its possible use as an excipient for the drugs [23]. Nanocomposites made by grinding carvacrol with Pal exhibited a good antibacterial property [24].

RT, the active ingredient of Zantac^®^, which is currently under recall by the US FDA as of April 2020, is a selective H_2_-receptor antagonist that can significantly inhibit gastric acid secretion and is prescribed for the treatment of peptic ulcers and related disorders [25]. Tests on its compatibility with different excipients, including talc, showed interactions over 3 months of storage for all excipients as confirmed by the TG and FTIR analyses [26]. Previous research on the sorption of RT by clay minerals suggested that the electrostatic interactions between the clay minerals and RT were responsible for its removal from water [8,20]. The goal of this study was to (1) evaluate the drug release from the mineral surfaces under different physicochemical conditions using RT as an example and (2) assess the applicability of using clay minerals as excipients for the manufacturing of RT. Most importantly, the results would add value through studying the interactions between Earth materials and pharmaceutical active ingredients, so that antagonistic effects would be avoided if Earth materials were to be used as inexpensive excipients for certain drug formulations in the pharmaceutical industry.

## 2. Materials and Methods

### 2.1. Materials

The selected excipients used were poorly crystallized 1:1 layered Kao (standard clay mineral KGa-2), fibrous clay mineral Pal (standard clay mineral PFl-1), and 2:1 layered talc. The first two were obtained from the Clay Mineral Repository in Purdue University (West Lafayette, IN, USA). The talc was purchased from Acros (Geel, Antwerp, Belgium). The SAA was 21.7 and 173 m^2^/g for Kao and Pal, respectively [27], and 2.3 m^2^/g for talc. The point of zero charge (pzc) was 4, 7.7, and 8 for Kao, talc, and Pal, respectively [10,28,29,30]. The Kao was almost pure, while the talc had about 2% clinochlore. In contrast, the Pal had about 80% Pal, 10% smectite, 7% quartz, 2% feldspar, and 1% other [31].

**Figure 1:**
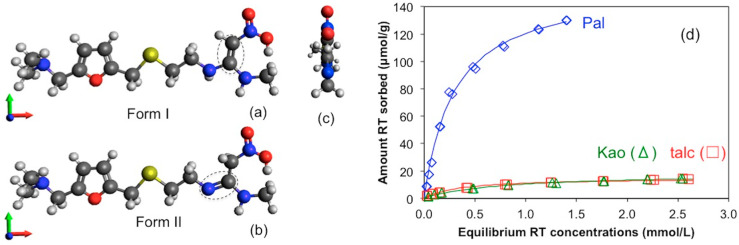
Molecular structure of ranitidine with (**a**) form I and (**b**) form II, and (**c**) their side view. The yellow is S, red is O, blue is N, dark gray is C, and the light gray is H. (**d**) Sorption of ranitidine (RT) on clay minerals.

The representative pharmaceutical compound was ranitidine (RT, or N-[2-[[[5-1[(dimethylamino)methyl]-2-furanyl]methyl]thio]ethyl]-N’-methyl-2-nitro-1,1-ethenediamine [32], in the HCl form. It was purchase from Alfa Aesar (Tewksbury, MA, USA). It may exist in a crystalline state as two tautomeric forms (structural isomers), form I or form II (Figure 1), each with its own crystal structure, melting point, and stability field [33]. It has a CAS# of 66357-59-3, a molecular mass of 350.86 g/mol, and an octanol–water partition coefficient close to 2 (logP∼0.3) [34]. It has two pKa values at 1.95 and 8.13, corresponding to the protonation of 2-nitroethene diamine and the dimethyl amino group [25]. Under an ambient pH it exists as a monovalent cation with the dimethyl amino group being protonated [25].

### 2.2. Sorption and Desorption Experiments

The uptake of RT on the clay minerals is strongly affected by their CEC values. As such, 1.0 g of Kao or talc, or 0.2 g of Pal, and 10 mL of RT aqueous solution were added to 50 mL centrifuge tubes. The initial RT concentrations varied from 0 to 4 mmol/L. The mixtures were shaken for 24 h at 150 rpm, and then centrifuged at 3500 rpm for 10 min. The supernatants were filtered through 0.45 μm syringe filters before being analyzed for equilibrium RT concentrations using a UV–VIS method. For the desorption experiments, 1.0 g of Kao or talc, or 0.2 g of Pal, and 10 mL of RT aqueous solution at a concentration of 1 mmol/L were mixed in each 50 mL centrifuge tubes. A concentrated RT stock solution was diluted by solutions of pH 6 and 11 or 1 mol/L NaCl in order to adjust the solution pH and ionic strength. After 24 h of mixing at 150 rpm, the mixtures were centrifuged at 3500 rpm for 10 min, and 8 mL of supernatants were removed followed by adding 8 mL of new solution. The mixtures were then shaken for 1, 2, 3, 4, and 5 h to investigate the desorption of RT under different solution pH and ionic strength conditions. The supernatants of each experimental condition were analyzed with the UV–VIS method.

### 2.3. Instrumental Analyses

The equilibrium solution RT concentrations were measured with the UV–VIS method at a wavelength of 312 nm, which is stable in a pH 3.5 to 12 range [25]. The powder X-ray diffraction (XRD) analyses were conducted using a D8 ADVANCE diffractometer (Bruker Corp., Billerica, MA, USA) under CuKα radiation at 40 kV and 40 mA. The samples were scanned from 2 to 40° 2*θ* with a scanning speed of 0.01°/s. The FTIR spectra were acquired from 650 to 4000 cm^−1^ by accumulating 256 scans at a resolution of 4 cm^−1^ using a Jasco FT/IR-4100 Spectrometer (Jasco, Jersey City, NJ, USA) equipped with a ZnSe crystal as the attenuated total reflection accessory.

## 3. Results

### 3.1. RT Sorption on the Excipients

The sorption of RT on the excipients is demonstrated in Figure 1d. Data were fitted to different sorption models and followed the Langmuir model best. It has the following formula:

(1) $$C_{s}=\frac{K_{L}S_{m}C_{L}}{{1+K}_{L}C_{L}}$$

and it can be converted into a linear form, as follows:

(2) $$\frac{C_{L}}{C_{s}}=\frac{1}{K_{L}S_{m}}+\frac{C_{L}}{S_{m}}$$

where C_S_ is the amount of RT sorbed at equilibrium (µmol/g), S_m_ the RT sorption capacity (µmol/g), C_L_ the equilibrium RT concentration (mmol/L), and K_L_ the Langmuir coefficient (L/mmol), reflecting the affinity of RT for the excipients. The fitted S_m_ values were 156, 18, and 15 µmol/g for RT uptake on Pal, Kao, and talc, respectively, while their SSA values were 173, 21.7, and 2.3 m^2^/g. As such, the RT sorption densities were 0.89, 0.83, and 6.52 µmol/m^2^ for the Pal, Kao, and talc, respectively.

The CEC values were 175 and 37 mmol_c_/kg for Pal and Kao, respectively [35]. Thus, for Pal, the RT sorption capacity was close to its CEC value, while for Kao, the RT sorption capacity was only 50% of its CEC value. In contrast, talc had essentially no permanent surface charges, because of its low isomorphous substitution in tetrahedral and octahedral sites [36]. In a study, the surface charge was reported as −3 µeq/g [37], far less than the RT sorption capacity of 15 µmol/g. Its extremely low CEC value originated from pH-dependent surface charges. In comparison, the RT sorption capacity on a diosmectite was 610 µmol/g [38]. Cation exchange was not deemed as a responsible mechanism for the uptake of AT on talc, nor the uptake of metoprolol (MT) on Kao and talc [7,39]. Thus, cation exchange is the most important mechanism for RT uptake on Pal. For Kao, cation exchange may play a partial role. In contract, the RT uptake on talc may be attributed to hydrophobic interactions, which may result in multi-layer sorption.

### 3.2. XRD Analyses

**Figure 2:**
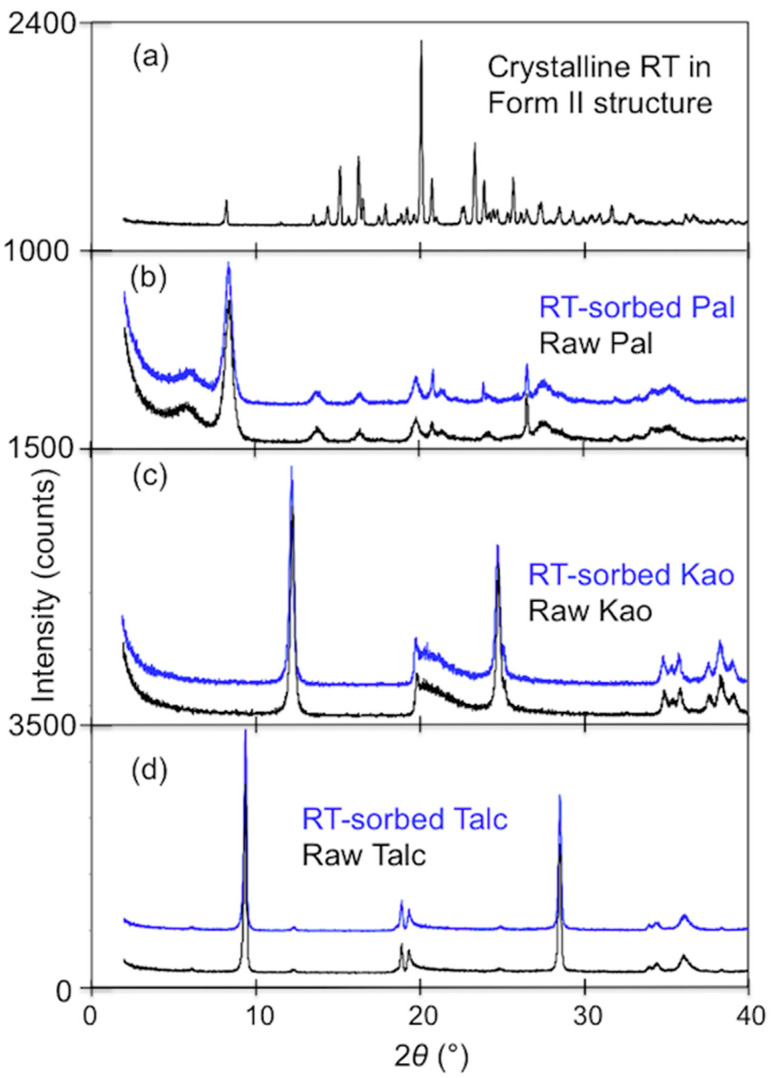
X-ray diffraction patterns of (**a**) crystalline RT, (**b**) Pal, (**c**) Kao, and (**d**) talc before and after RT sorption.

The XRD of the crystalline RT (Figure 2) confirmed that the sample used was in form II, which had 2*θ* values of 20.0, 23.3, and 27.4° for the three strongest peaks [33]. The XRD patterns of Pal, Kao, and talc before and after RT uptake showed no changes in the locations nor the intensities of the strong diffraction peaks, suggesting that the uptake of RT by these excipients was on external surfaces (Figure 2). In comparison, the RT uptake on MMT involved in intercalation as confirmed by the d_001_ spacing expansion to 16.8 Å [40]. In addition, no RT peaks were found on the XRD patterns of Pal, Kao, and talc, indicating the sorption of RT on these excipients instead of the RT precipitation from solution.

### 3.3. Kinetics of RT Desorption from the Excipients

**Figure 3:**
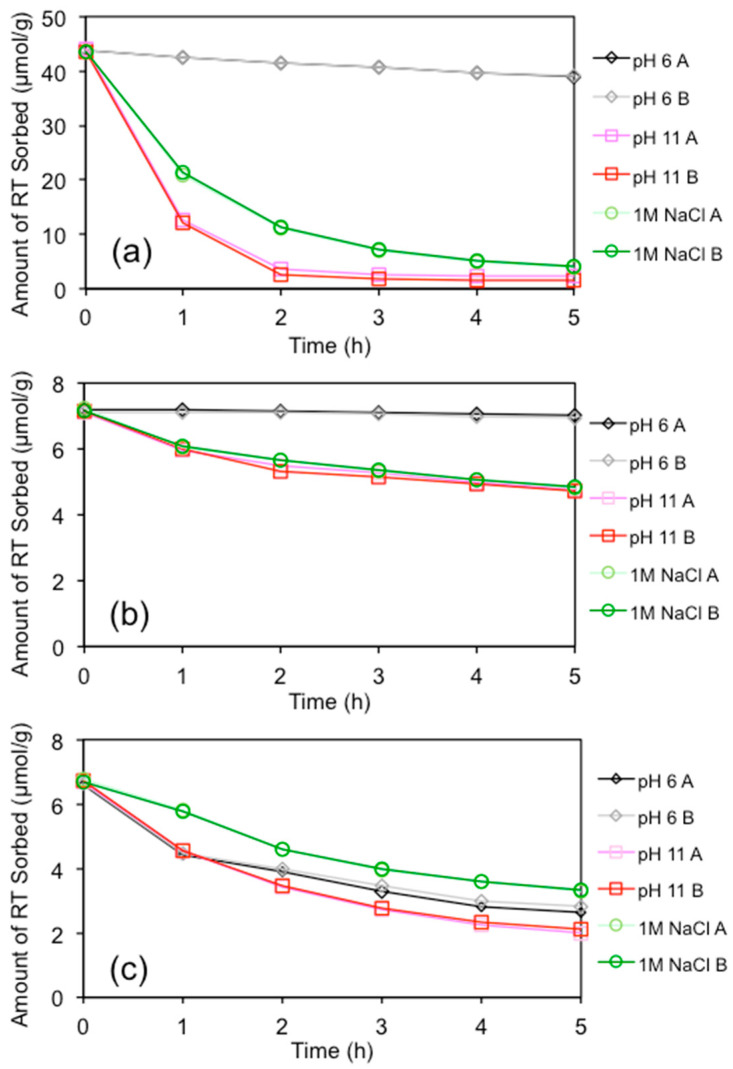
Desorption of RT from (**a**) Pal, (**b**) Kao, and (**c**) talc.

The initial uploads of RT on Pal, Kao, and talc were 44, 7.2, and 6.8 µmol/g, respectively. For Pal, at an equilibrium solution pH of 6, the amount of RT desorbed after 5 h was 4.8 µmol/g, accounting for slightly over 10% (Figure 3). However, under an equilibrium solution pH of 11 and under an ionic strength of 1.0 M NaCl, 90–95% of the initially sorbed RT desorbed from the mineral surfaces. For Kao, negligible RT desorption occurred at pH 6, but about 30% RT was desorbed using solutions of pH 11 and an ionic strength of 1.0 M NaCl after 5 h of desorption. In comparison, about 50–60% sorbed RT desorbed from talc surfaces under pH 6, pH 11, and 1.0 M NaCl conditions. The difference may be attributed to the bonding between RT and the excipients, and the surface configuration of the sorbed RT molecules on these excipients. For Pal, it is a type of fibrous clay mineral, while the other two are platy. Thus, the sorption sites of RT on Kao and talc are on the tetrahedral or octahedral sheets. For Pal, the uneven surface may make the sorbed RT molecules susceptible to desorption. As the pKa values of RT are 1.95 and 8.13 [25], and it exists as a monovalent cation at pH 6 and in a 1 M NaCl solution, but in neutral species under pH 11. The speciation played a significant role in the RT desorption, suggesting that maximal RT sorption could be achieved in the intestinal fluid when Pal and Kao are used as excipients.

No reports on RT desorption from the mineral excipient surfaces were reported. However, conflicting results were found in the literature regarding RT sorption on solids being affected by the solution pH. In one study, RT sorption on smectite was found to be independent of the solution pH [41]. In another study, the RT uptake increased as the solution pH increased [38]. More recently, maximum RT intercalation in the interlayer of MT was found at pH 4–8 [40]. Previous results showed that the uptake of RT on talc was less sensitive in the equilibrium solution pH, and the amount of RT uptake was 6 and 4 µmol/g at pH 6 and 11, respectively [8]. On the other hand, the RT uptake on Kao and Pal dropped from 12 to 1 µmol/g and from 55 to less than 10 µmol/g, respectively, when changing from pH 6 to pH 11 [8,20]. As RT is a neutral molecule at pH 11, the electrostatic interactions between RT and Kao or Pal surfaces decreased drastically. As such, the significant desorption of RT at pH 11 may indicate that the initial uptake of RT on Kao and Pal was via an electrostatic interaction.

### 3.4. Molecular Simulation

**Figure 4:**
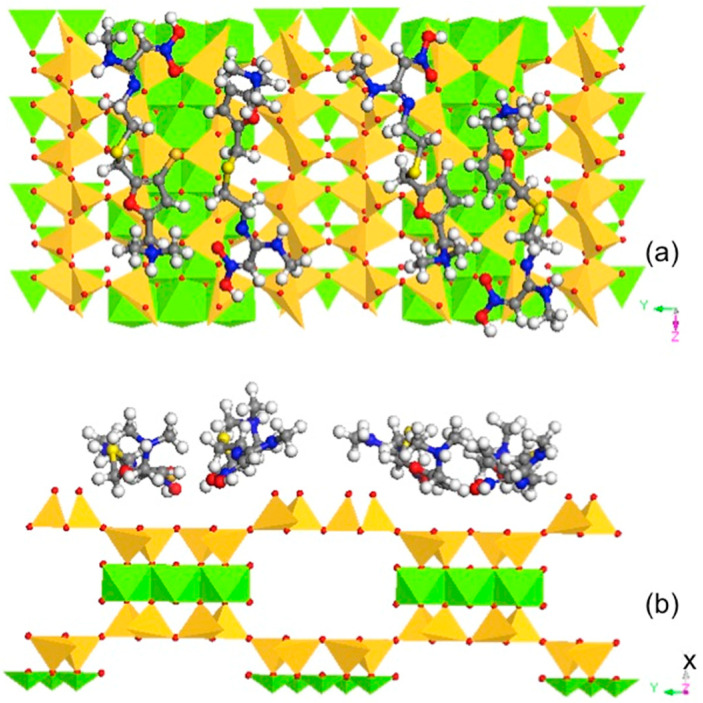
Molecular dynamic simulation showing the monolayer surface configuration of sorbed RT on Pal along (**a**) [100] and (**b**) [001] directions.

For Pal, using an SSA of 173 m^2^/g [27] and an RT sorption capacity of 156 µmol/g, the calculated space per RT molecule occupied was 1.84 nm^2^. The unit cell of Pal was *a* = 1.278, *b* = 1.786, and *c* = 0.524 nm [42]. As the 2:1 layer was perpendicular to the *a* direction, assuming that the uptake of RT was on the 2:1 layer, the simulation was performed on the *bc* plane using 2*b* × 4*c*, which had an area of 7.49 nm^2^. Thus, four RTs were used. The simulation results showed a compact monolayer RT configuration with the molecules lying nearly flat on the surface of the Pal (Figure 4). Thus, taking into consideration the CEC and SSA values, both are limiting factors for RT sorption on Pal.

**Figure 5:**
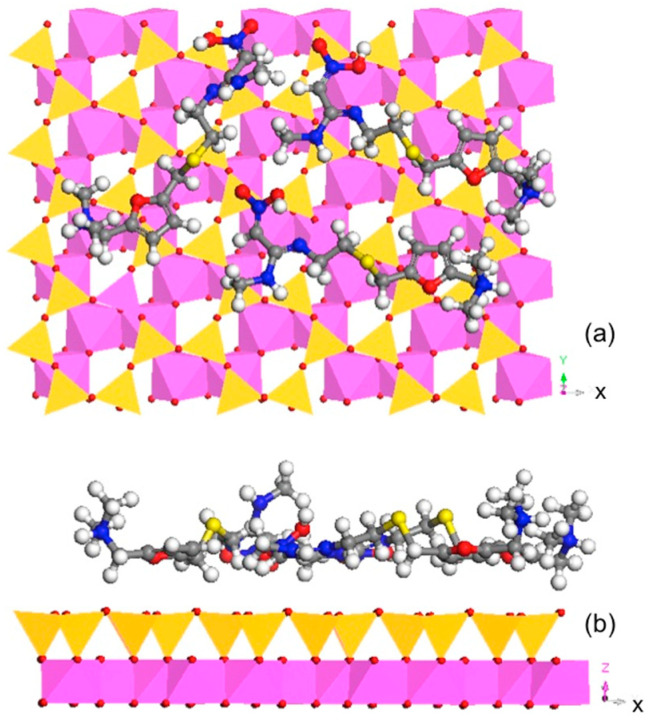
Molecular dynamic simulation showing the monolayer surface configuration of sorbed RT on Kao along (**a**) [001] and (**b**) [010] directions.

For Kao, using an SSA of 21.7 m^2^/g [27] and an RT sorption capacity of 18 µmol/g, the calculated space per RT molecule occupied was 2.00 nm^2^. The unit cell of Kao was *a* = 0.515, *b* = 0.895, and *c* = 0.740 nm [43]. As the 1:1 layer was perpendicular to the *c* direction, the simulation was performed on the *ab* plane using 4*a* × 3*b*, which had an area of 5.48 nm^2^. Thus, three RTs were used. Again, the simulation results showed an almost compact monolayer surface configuration of RT on the tetrahedral sheet of Kao, which is where the seat of charge originated (Figure 5). On the other hand, the RT sorption was only 50% of the CEC value of Kao. Thus, for Kao, the SSA value might be the limiting factor for monolayer sorption.

**Figure 6:**
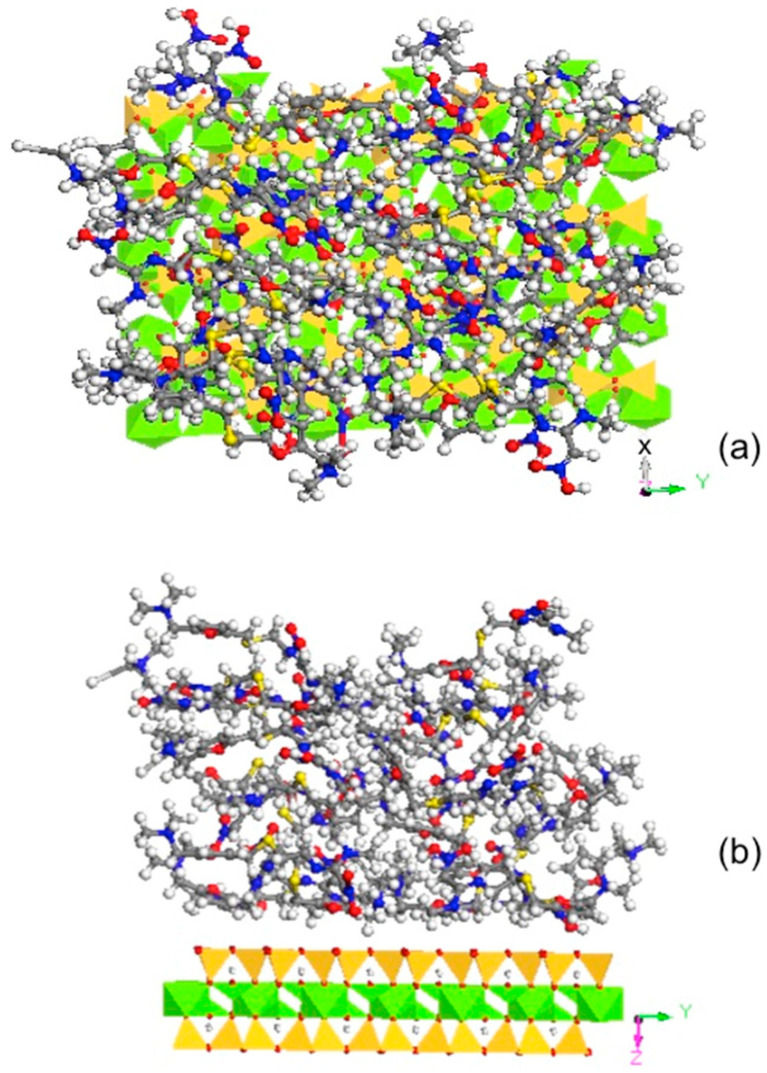
Molecular dynamic simulation showing the multilayer surface configuration of sorbed RT on talc along (**a**) [001] and (**b**) [100] directions.

Similarly, for talc, an SSA of 2.3 m^2^/g and an RT sorption capacity of 15 µmol/g were used. The calculated space per RT molecule occupied was 0.25 nm^2^. The unit cell of talc was *a* = 0.529, *b* = 0.918, and *c* = 0.950 nm [44], and the 2:1 layer was perpendicular to the *c* direction. The simulation was performed on the *ab* plane using 4*a* × 3*b*, which had an area of 5.83 nm^2^. Thus, 23 RT molecules were used. In this case, the sorbed RT molecules formed a multilayer on the surface of the talc (Figure 6). As the critical micelle concentration of RT is 1 × 10 ^−6^ M [45], the sorbed RT may also be considered in admicelle forms. As talc is a trioctahedral 2:1 layered phyllosilicate without a swelling property, with limited SSA values, and is hydrophobic, the sorbed RT would interact with the talc surface and with each other via hydrophobic interactions.

### 3.5. FTIR Analyses

**Figure 7:**
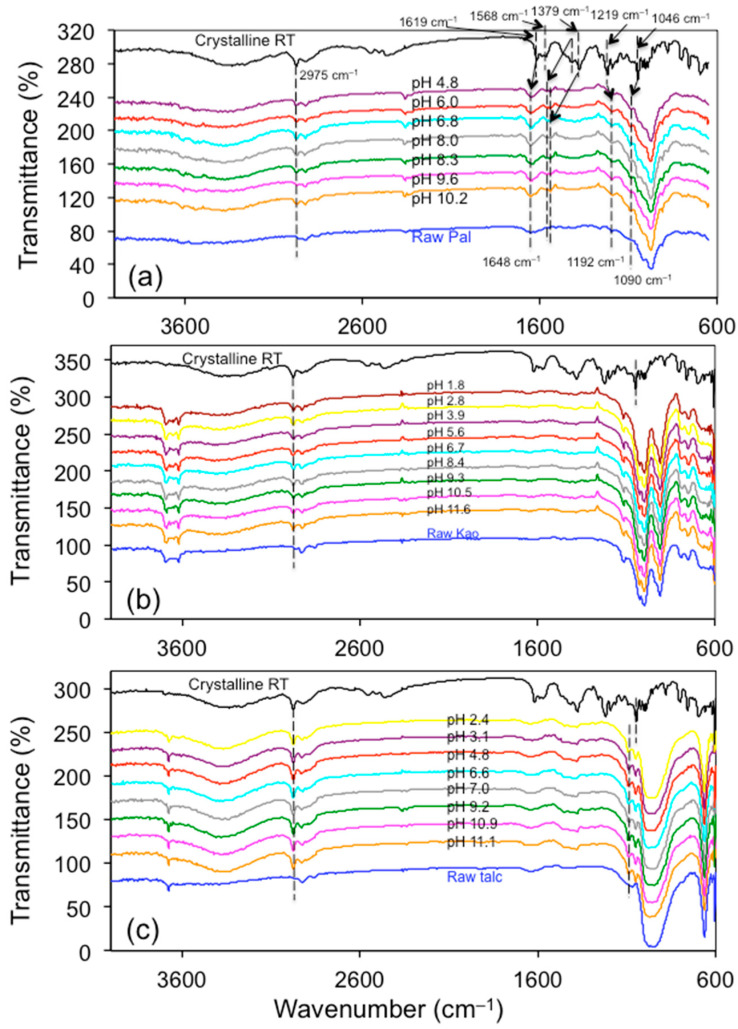
FTIR spectra of the RT loaded Pal (**a**), Kao (**b**), and talc (**c**) under different equilibrium solution pHs.

The two tautomeric forms of RT hydrochloride differed considerably in detail, especially in the region above 3000 cm^−1^ (bonded NH absorption), as well as in the complex peaks of the protonated dimethylamino group in the 2700–2300 and 1620–1570 cm^−1^ regions [46]. The FTIR spectrum of the crystalline RT (Figure 7) matched well with that of form II [32,46,47], confirming the XRD results.

The amount of RT sorbed on Pal was 44 µmol/g, or 15 mg/g. For the raw Pal, the band at 2975 cm^−1^ was missing, but for the other Pal samples desorbed under different solution pHs, it was present (Figure 7a). The bands at 1619 and 1568 cm^−1^ were assigned to the N–H bond [48]. These bands shifted to 1648 cm^−1^ and turned into just one band. Perhaps this might be due to protonation of the N–H to N–H_2_^+^, which may interact with the negatively charged mineral surfaces through electrostatic interactions. The band at 1610 cm^−1^ was assigned to the C–N bond in the plane bending of the amino groups [47]. It was located at 1619 cm^−1^ in this study and shifted to 1648 cm^−1^, suggesting the participation of amino groups for the electrostatic interaction between RT and Pal.

The band at 1379 cm^−1^ was assigned to the ν_as_ (NO_2_) group [49], and it disappeared after RT sorption on Pal. The band at 1240 cm^−1^ was assigned to the C–N bond characteristic of a nitro group attached to a substituted ethene group [32]. In this study, it was located in 1219 cm^−1^ and it shifted to 1192 cm^−1^ after the RT sorption on Pal. The bands at 1015 cm^−1^ were assigned to a 2,5-disubstituted furan [46]. In this study, it was located at 1046 cm^−1^ and it shifted to 1090 cm^−1^ after sorption on Pal. A high positive electrostatic potential was found around the furan ring of RT [50]. These results suggest that the amide and furan groups would be more likely to be involved in the interactions between RT and Pal.

For Kao and talc, most of the RT bands did not show up (Figure 7b,c). This is because the amount of RT loading was 7.2 and 6.8 µmol/g, corresponding to 2.5 mg/g, or 0.25% of the mass of the solid, respectively. Thus, it was not surprising that most of the RT bands were not resolved. Still, the band at 2975 cm^−^^1^ was present for the RT sorbed Kao and talc, confirming the presence of RT on these minerals. For talc, the band at 1619 cm^−^^1^ was also present on the RT sorbed samples. It has been suggested that the interactions between talc and RT were via the NO_2_ group and the 2.5-disubstituted furan through a delocalized π-bond [8].

## 4. Discussion

RT is a nearly flat molecule with a dimension of 1.73 nm long by 0.55 nm wide by about 0.3 nm thick, resulting in a surface area of 1.0 nm^2^ for a flat lying orientation. Thus, with an SSA of 2.3 m^2^/g for talc and a loading of 6.8 µmol/g before desorption, the area occupied per RT molecule on the talc surface would be 0.55 nm^2^. The XRD results showed no peak shifting nor peak broadening, suggesting no interlayer sorption nor breakdown of the talc particle size. As such, the multi-layer RT sorption on talc was speculated and confirmed by the molecular simulations. On the other hand, at the RT sorption of 7.2 and 44 µmol/g on Kao and Pal, the available space for each sorbed RT molecule would be 5.0 and 6.5 nm^2^, respectively. Thus, a less condense monolayer sorption was anticipated.

As these minerals were used either as active ingredients or excipients, the uptake of RT by these minerals, particularly Pal, may signal an antagonistic effect in the process of mixing or in the stomach under stomach fluid mixing, if Pal and RT were taken simultaneously or used together in a pharmaceutical formulation. Moreover, the small amounts of RT released under neutral and slightly alkaline conditions, and under elevated ionic strength conditions, also support strong bonding between the RT and Kao. Thus, the results from this study suggest that future studies on clinical effects should be initiated.

## 5. Conclusions

In this study, the uptake and release of RT on or from the selected clay minerals as excipients were assessed under different physicochemical conditions, and were characterized using instrumental analyses and molecular dynamic simulation. The results showed that the uptake of RT on the excipients was affected by the SSA and CEC values of the excipients. Different types of interactions affected the release of RT from the excipient surfaces. Thus, care must be taken when formulating active ingredients that are cationic in nature with negatively charged clay minerals as excipients, so that the proper release of active ingredients would be warranted in order to achieve the effectiveness of the active ingredients. For the cationic drugs, when the pH is greater than the pKa value of the drugs, a better release from the excipient surface could be achieved. As such, in order to achieve intestinal RT sorption, Pal may be a better choice for excipients.
